# Utilization of hematopoietic stem cell transplantation for the treatment of multiple myeloma: a Mayo Stratification of Myeloma and Risk-Adapted Therapy (mSMART) consensus statement

**DOI:** 10.1038/s41409-018-0264-8

**Published:** 2018-07-09

**Authors:** Wilson I. Gonsalves, Francis K. Buadi, Sikander Ailawadhi, P. Leif Bergsagel, Asher A. Chanan Khan, David Dingli, Angela Dispenzieri, Rafael Fonseca, Susan R. Hayman, Prashant Kapoor, Taxiarchis V. Kourelis, Martha Q. Lacy, Jeremy T. Larsen, Eli Muchtar, Craig B. Reeder, Taimur Sher, A. Keith Stewart, Rahma Warsame, Ronald S. Go, Robert A. Kyle, Nelson Leung, Yi Lin, John A. Lust, Stephen J. Russell, Stephen R. Zeldenrust, Amie L. Fonder, Yi L. Hwa, Miriam A. Hobbs, Angela A. Mayo, William J. Hogan, S. Vincent Rajkumar, Shaji K. Kumar, Morie A. Gertz, Vivek Roy

**Affiliations:** 1 Division of Hematology, Mayo Clinic, Rochester, Minnesota USA; 2 Division of Hematology and Medical Oncology, Mayo Clinic, Jacksonville, Florida USA; 3 Division of Hematology and Medical Oncology, Mayo Clinic, Scottsdale, Arizona USA

**Keywords:** Chemotherapy, Myeloma

## Abstract

Over the last two decades, the utilization of various novel therapies in the upfront or salvage settings has continued to improve survival outcomes for patients with Multiple Myeloma (MM). Thus, the conventional role for hematopoietic stem cell transplantation (HSCT) in MM either in the form of an autologous stem cell transplant (ASCT) or an allogeneic stem cell transplant (Allo-SCT) warrants re-evaluation, given the aforementioned clinical advances. Here, we present a consensus statement of our multidisciplinary group of over 30 Mayo Clinic physicians with a special interest in the care of patients with MM and provide evidence-based recommendations on the use of HSCT in MM. We specifically address topics that include the role and timing of an ASCT for MM in the era of novel agents, eligibility for an ASCT, post-ASCT consolidation, and maintenance options, and finally the utility of an upfront tandem ASCT, salvage second ASCT, and an allo-SCT in MM.

## Introduction

Hematopoietic stem cell transplantation (HSCT) involves high-dose chemotherapy followed by infusion of procured autologous, allogeneic, or syngeneic stem cells. Utilization of autologous stem cell transplant (ASCT) has increased in the United States and Europe over the last decade and is most commonly used for the treatment of multiple myeloma (MM) [1, 2]. The therapeutic armamentarium for MM has evolved over the last two decades with the incorporation of numerous novel therapies such as proteasome inhibitors (PIs), immunomodulators (IMiDs), and monoclonal antibodies (mABs) for the treatment of patients with newly diagnosed and relapsed MM [3, 4]. This has led to an improvement in the depth and duration of disease response that have eventually translated to an improved overall survival (OS) for patients with MM [5, 6]. Given that the therapeutic regimens and their utilization have evolved dramatically in clinical practice, the current role of HSCT in MM at all stages of the disease warrants a systematic re-evaluation. Our multidisciplinary group of over 30 Mayo Clinic physicians at three different sites (Scottsdale, AZ; Jacksonville, FL; and Rochester, MN) with a special interest in the care of patients with MM have performed an extensive review of the literature with the aim of making evidence-based recommendations on the use of HSCT for the management of patients with MM. This is part of the Mayo Stratification for Myeloma and Risk-Adapted Therapy (mSMART) guidelines that are available at http://www.msmart.org and updated regularly in light of new data. A standard system for rating the evidence and grading of recommendations will be utilized as outlined in Table 1. Emphasis is placed on the outcomes from randomized controlled trials (RCTs), but in the absence of such data, recommendations are based on other non-RCT data and consensus within the group. Given that this article relates only to the utilization of HSCT for the treatment of MM, we will not discuss the diagnosis and initial management of the disease or other treatment options for relapsed disease. The reader is referred to various guidelines that have been published by our group in this regard [3, 4, 7, 8].

**Table 1:** Classification system for levels of evidence and grades of recommendations

Level	Type of evidence
I	Evidence obtained from a meta-analysis of multiple, well-designed, controlled studies. Randomized trials with low false-positive and low false-negative errors (high power)
II	Evidence obtained from at least 1 well-designed experimental study. Randomized trials with high false-positive and/or false-negative errors (low power)
III	Evidence obtained from well-designed, quasi-experimental studies such as non-randomized, controlled single-group, pre–post, cohort, time series, or matched case-control series
IV	Evidence from well-designed, non-experimental studies, such as comparative and correlational descriptive and case studies
V	Evidence from case reports and clinical samples
Grade	Type of evidence
A	Evidence of type I or consistent findings from multiple studies of type II, III, or IV
B	Evidence of type II, III, or IV, and findings are generally consistent
C	Evidence of type II, III, or IV, but findings are inconsistent
D	Minimal or no systematic empirical evidence

## Rationale for the use of ASCT in the novel agent era

Utilization of high-dose intravenous melphalan chemotherapy for the treatment of MM was first described by McElwain and Powles in 1983 [9]. Subsequent studies ameliorated the myelosuppressive effects of the high-dose melphalan with a subsequent autologous hematopoietic stem cell infusion [10, 11]. However, ASCT became the standard of care for MM only after positive results from phase III RCTs (Supplement Table 1) demonstrated the superiority of ASCT compared to conventional cytotoxic chemotherapy by improving the depth as well as duration of hematological response [12–17]. All trials except for one demonstrated improvement in the depth of hematologic response and progression-free survival (PFS) or event-free survival (EFS) favoring the use of an ASCT. However only half of the trials, including two of the largest trials, demonstrated significant improvements in OS of almost 12 months with ASCT [12, 13]. A meta-analysis of all these earlier trials, which were done prior to the availability of IMiDs or PIs, supported the use of ASCT in terms of PFS extension but not OS [18]. Furthermore, one trial also demonstrated a better QoL for patients undergoing an ASCT which was defined as a significantly longer period of time without symptoms, treatment, and treatment toxicity (TwiSTT) [19]. Thus, based on these positive benefits associated with ASCT as consolidative therapy, it was incorporated as the standard of care for eligible MM patients.

Currently, PI and/or IMiD combination regimens are considered the standard of care for induction therapy as they have consistently led to deeper and more durable hematologic responses in almost all MM patients at all phases of the treatment (induction, consolidation, and maintenance). As a result, the value of ASCT as a standard of care for all eligible MM patients has been questioned, especially since the OS benefit has not been consistently noted in prior RCTs. Thus, it is important to assess all the existing phase III RCTs that evaluated the value of ASCT in the era of novel agent in;duction therapies, which are summarized in Table 2. Two RCTs utilized lenalidomide and dexamethasone induction followed by consolidation with either a tandem ASCT using high-dose melphalan as the conditioning regimen or a novel agent-based regimen (melphalan, prednisone, and lenalidomide (MPR) or cyclophosphamide, lenalidomide and dexamethasone (CRd)) [20, 21]. In a pooled analysis of both studies, significant improvements in PFS (42 months vs. 24 months) and a benefit in OS at a 4-year follow-up (84% vs. 70%) was observed in the groups undergoing ASCT as consolidation [22]. Even though most patients received two courses of melphalan 200 mg/m^2^ in tandem fashion in these trials, there was no difference in OS outcomes between those who completed only one ASCT versus those who received the intended tandem ASCT. An interim analysis from the European Myeloma Network Trial (EMN02) demonstrated that upfront consolidation with ASCT after bortezomib, cyclophosphamide, and dexamethasone induction was associated with a significant improvement in depth of response and median PFS compared to consolidation with bortezomib, melphalan, and prednisone (VMP) in the overall patient population (not reached vs. 44 months) [23]. This superiority of ASCT over VMP was even more prominent in the high-risk cytogenetics group in terms of 3-year PFS (52% vs. 30%) and 3-year OS benefit (74% vs. 61%) [23].

**Table 2:** Summary of phase III clinical trials evaluating the efficacy of ASCT in the era of novel agent induction therapy

Clinical trial	No.	Induction	Randomized arms	ORR	EFS/PFS	OS
Palumbo et al. [20]. NDMM pts <65 y/o	402	Rd × 4 cycles	MPR × 6 cycles →No maint	CR (post consolidation): 18%	Median PFS: 22 mos	5-year OS: 59%
			MPR × 6 cycles →R-maint		Median PFS: 34 mos	5-year OS: 70%
			Mel200 mg/m^2^ × 2 cycles → No maint	CR (post consolidation): 23%	Median PFS: 37 mos	5-year OS: 67%
			Mel200 mg/m^2^ × 2 cycles → R-maint		Median PFS: 55 mos	5-year OS: 78%
Gay et al. [21]. NDMM pts ≤65 y/o	389	Rd × 4 cycles	CRd × 6 cycles → R-maint	CR: 27%	Median PFS: 28 mos	4-year OS: 76%
			CRd × 6 cycles → Rp-maint	CR: 23%	Median PFS: 24 mos	4-year OS: 68%
			Mel200 mg/m^2^ × 2 cycles → R-maint	CR: 33%	Median PFS: 32 mos	4-year OS: 75%
			Mel200 mg/m^2^ × 2 cycles → Rp-maint	CR: 37%	Median PFS: 38 mos	4-year OS: 77%
Cavo et al. [23]. NDMM pts ≤65 y/o	1503	VCd × 3–4 cycles	VMP→+/− VRd → R-maint	≥VGPR: 75%	3-year PFS: 57%	Median OS: NR
			Mel200 mg/m^2^ × 1 or 2 →+/− VRd →R-maint	≥VGPR: 84%	3-year PFS: 64%	Median OS: NR
Attal et al. [25]. NDMM pts ≤65 y/o	700	VRd × 3 cycles	VRd × 5 cycles →R-maint	CR: 48% MRD (−): 65%	Median PFS: 36 mos	4-year OS: 82%
			Mel200 mg/m^2^ →VRd × 2 cycles →R-maint	CR: 59% MRD (−): 79%	Median PFS: 50 mos	4-year OS: 81%

*CRd* cyclophosphamide, lenalidomide, and dexamethasone, *R-maint* lenalidomide maintenance, *Rp*-*maint* lenalidomide and prednisone maintenance, *VRd* bortezomib, lenalidomide, and dexamethasone, *MPR* melphalan, prednisone and lenalidomide, *VCd* bortezomib, cyclophosphamide and dexamethasone, *ORR* overall response rate, *EFS* event-free survival, *PFS* progression-free survival, *OS* overall survival, *CR* complete remission, *MRD* (−) minimal residual disease negativity

One drawback of all these prospective studies is the lack of PI and IMiD combination-based induction therapies especially since this combination is now considered to be the optimal induction regimen due to deeper and more durable responses than induction regimens with either PI or IMiDs alone [24]. The IFM/DFCI trial utilized bortezomib, lenalidomide, and dexamethasone (VRd)-based induction therapy with either an upfront or delayed consolidation with ASCT. Based on initial data from the IFM portion of the study, despite the use of a very effective induction regimen with VRd, ASCT consolidation and maintenance lenalidomide was superior in producing deeper hematologic responses, including more minimal residual disease (MRD) negative responses (79% vs. 65%, *P* <0.001) when compared to continued consolidation with VRd followed by maintenance lenalidomide [25]. At 4 years follow-up, patients treated with ASCT consolidation also had a superior PFS (50 months vs. 36 months) though no difference in 4-year OS was identified (81% vs. 82%). However, 79% of the patients who had disease progression on the non-ASCT arm eventually underwent a salvage ASCT [25].

Finally, a meta-analysis incorporating both conventional and network meta-analysis of three large phase III RCTs from January 2000 to April 2017 showed that consolidation with an ASCT was associated with superior PFS compared with therapy using novel agents (IMiDs and PIs) [26]. Thus, irrespective of the novel agent combination used in the induction therapy, it appears that ASCT as consolidation consistently improves the depth of response and PFS producing very good survival results. This reinforces the practice of ASCT as the standard of care for eligible patients with NDMM treated with novel agent therapies.

**Recommendation:** *In the era of novel agent induction therapy regimens, autologous stem cell transplant (ASCT) remains an essential component of MM therapy in eligible patients*. (**Level of Evidence:** I, **Grade of Recommendation:** A)

## Timing of ASCT: upfront versus upon first relapse

Given the likelihood of deep and durable hematologic responses obtained with either PIs and/or IMiDs-based induction therapy and the frequent but mostly transient toxicities associated with ASCT, the timing of an ASCT during the course of a patient’s MM has been debated, i.e., should all eligible patients undergo an upfront ASCT as consolidation after initial induction therapy or can ASCT be delayed until first relapse? Though the first phase III RCT to assess this question was conducted in the pre-novel agent era, it demonstrated that despite equivalent survivals, patients undergoing an upfront ASCT experienced a longer period of time free from treatment and consequent side effects than patients who continued on chemotherapy [19]. This advantage is less applicable to current practice given the almost universal use of continued maintenance therapy after an upfront ASCT [27]. The current drug regimens are also associated with less toxicity compared with the older alkylator-containing regimens allowing for longer treatment durations. Prior single institution retrospective studies have suggested that early ASCT (within 12 months of diagnosis) provided superior PFS but similar OS compared to patients who underwent delayed ASCT in the setting of novel agent induction therapy [28, 29]. The IFM portion of the IFM-DFCI study, after a median follow-up of 39 months, demonstrated that the use of an upfront ASCT improved the depth of hematologic response both in terms of rates of CR (59% vs. 48%) as well as MRD negativity (79% vs. 65%) in patients who obtained at least a very good partial response (VGPR). An upfront ASCT also improved median PFS (50 months vs. 36 months) when compared to a delayed ASCT upon first relapse [25]. In addition, QoL assessments favored the use of an upfront ASCT. However, the 3-year post-randomization rate of OS was similar in the two groups [25].

**Fig. 1:**
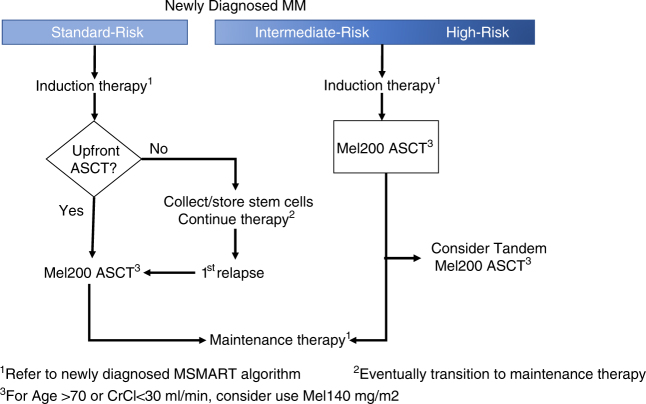
The mSMART algorithm for utilization of ASCT for the treatment of MM in newly diagnosed patients based on cytogenetic risk

Thus, given that most studies have demonstrated an improvement in depth of response and PFS with upfront ASCT, we recommend that it should be the standard approach for eligible MM patients. However, given the equivalent OS with either upfront or delayed ASCT strategies, delaying an ASCT due to personal choice or other logistical situations is reasonable especially in standard-risk patients. This should be done in the context of a thorough discussion with each patient informing them about the risk of not being suitable candidates for ASCT in the future upon first disease relapse. A retrospective study from our institution estimated this risk at about 10% either due to poor performance status, worsening comorbidities or rapid uncontrolled disease relapse [30]. In contrast, for patients with high-risk disease by cytogenetics or gene expression profiling, in the absence of participation in a clinical trial, our practice at the Mayo Clinic is to recommend the use of an upfront ASCT over a delayed ASCT given that the best OS outcomes to date for this patient population seems to be derived from phase III clinical trials in which they all underwent an upfront ASCT [31]. Early ASCT offers the best chance of achieving CR, especially MRD-negative status which has been shown to be associated with better survival outcomes [32, 33]. Finally, it is important to reiterate that the RCTs have shown equivalence in OS between upfront ASCT and ASCT upon first relapse and not upfront ASCT versus no ASCT ever or ASCT beyond second line therapy. Fig. 1 summarizes the mSMART algorithm for utilization of ASCT for the treatment of MM in newly diagnosed patients based on cytogenetic risk.

**Recommendation:** *Though a delayed ASCT upon first relapse is safe and feasible, an upfront ASCT in eligible MM patients remains the standard of care especially in those patients with high-risk disease*. (**Level of Evidence:** I, **Grade of Recommendation:** A)

## Eligibility for ASCT

Most clinical trials assessing the efficacy and safety of ASCT in MM enrolled patients younger than 65 years and this cut-off remains the standard inclusion criterion in most European countries. However, several studies have demonstrated the feasibility of performing an ASCT in MM patients 65 to 76 years of age with similar efficacy and toxicities to younger patients [34–36]. However, co-morbidities do affect the outcomes of ASCT. Retrospective registry data have suggested that the HSCT co-morbidity index (HCT-CI) score typically used for allo-SCTs, where the higher score is associated with higher morbidity, can predict for worse outcomes in patients with MM undergoing ASCT [37]. Careful patient selection based on overall health status rather than just chronological age or presence of renal insufficiency is important to ensure an optimal balance between risks and benefits.

**Recommendation:** *In contrast to strict chronologic age cutoffs for ASCT eligibility, performance status and comorbidities should be considered for ASCT eligibility*. **(Level of Evidence:** III, **Grade of Recommendation:** B)

In general, depth of response to initial induction chemotherapy has been positively associated with outcomes post-ASCT [38]. However, it should be noted that very rapid and deep responses to induction therapy in some patients with MM have been subsequently followed by early and quick disease relapses [39]. Thus, depth of response is not always predictive of a positive outcome. A retrospective study from the International Bone Marrow Transplant Registry in the US showed no OS benefit when providing additional induction therapy to deepen the hematological response in MM patients with at least a partial response (PR) to the original induction therapy [40]. Furthermore, several studies have evaluated the use of ASCT in patients with refractory MM that show even such patients may benefit from an upfront ASCT though this was limited to pre-novel agent era studies [41–44]. Current upfront MM therapy typically does not include conventional cytotoxic chemotherapy. So, a poor response to these novel agents does not necessarily preclude a response to alkylator-based therapy such as high-dose melphalan followed by an ASCT. Recent retrospective studies suggest that even patients with refractory disease to novel agent regimens can gain therapeutic benefit from ASCT though less than those with responsive disease [45]. Thus, patients not responding to initial induction therapy will likely experience worse OS since they have a more chemoresistant tumor. Such patients should undergo an ASCT as soon as possible rather than delay the process for further tumor debulking with salvage chemotherapy. The MRC XI phase III trial evaluated the value of deepening hematological responses prior to ASCT in a prospective randomized fashion by providing additional sequential cycles of bortezomib, cyclophosphamide, and dexamethasone to half of the patients who experienced a suboptimal hematologic response (achieving only an MR or PR) with IMiD-based triplet induction therapy [46]. This additional PI-based therapy upgraded the hematological responses from MR or PR to a VGPR or better in 41% of evaluable patients. Patients who received additional sequential cycles of PI-based therapy prior to an ASCT had an improved median PFS of (55 vs. 30 months, *P* = 0.0003) but no difference in OS when compared to those patients who did not receive any further therapy prior to proceeding to an ASCT [46].

**Recommendation:** *Depth of response to induction therapy should not dictate eligibility for an ASCT*. (**Level of Evidence:** III, **Grade of Recommendation:** B)

## Stem cell mobilization

Direct harvesting of hematopoietic stem cells from the bone marrow is rarely performed for ASCTs and instead mobilization of CD34+ cells from the bone marrow compartment to the peripheral blood is the mainstay of collecting hematopoietic stem cells. The peripheral blood approach to stem cell mobilization can be performed with the use of growth factor alone, growth factor in combination with cytotoxic chemotherapy “chemomobilization” or growth factor in combination with plerixafor. The ideal approach to mobilize stem cells should produce the highest yield with the lowest toxicity and cost for patients with MM [47]. The minimum number of peripheral blood stem cells (PBSCs) required for an ASCT is 2 × 10^6^ CD34+ cells/kg while 3 to 5 × 10^6^ CD34+ cells/kg are ideal [48]. Mobilization with G-CSF alone followed by initiation of apheresis is widely used and well tolerated. Plerixafor, a chemokine receptor 4 (CXCR4) antagonist which disrupts the interactions between the stromal derived factor 1 (SDF-1) and CXCR4, can enhance stem cell mobilization and yield from each apheresis session. Peripheral blood CD34+ cell count monitoring prior to apheresis can identify patients at risk of stem cell mobilizing failure [49]. In such cases, the use of plerixafor prior to apheresis triggered by the peripheral blood CD34+ cell count or initial apheresis yield can minimize the risk of stem cell mobilization failures [50]. Chemomobilization is especially useful in those patients with a high risk of stem cell mobilization failure as it can increase the PBSC yield while concomitantly decreasing the MM tumor burden. However, in a non-randomized assessment, this has not yielded superior OS outcomes compared to mobilization with G-CSF alone [51]. In addition, chemomobilization increases the risk of toxicity such as infections and is less predictable in timing to stem cell goal collection when compared to growth factor in combination with plerixafor [52, 53]. For these reasons, the role of chemomobilization has been questioned in the era of novel agent induction therapy [54]. Nevertheless, it can be considered in selected settings—prior failed stem cell mobilization with G-CSF and/or plerixafor, the presence of high numbers of circulating plasma cells in the peripheral blood, or in the presence of progressive disease at the time of stem cell mobilization [55].

Prolonged exposure to lenalidomide therapy has been associated with suboptimal CD34+ stem cell mobilization [56–58]. Fortunately, the use of plerixafor and/or chemomobilization with cyclophosphamide and G-CSF has been able to overcome the issue of poor mobilization related to prior lenalidomide exposure [59–63]. Thus, we recommend pursuing stem cell mobilization and collection within 4–6 cycles of lenalidomide exposure in all ASCT eligible patients even if planning for a delayed ASCT. Finally, salvage second and third ASCTs have been performed with considerable success in patients with relapsed MM [64]. In order to preserve these options of performing a tandem ASCT or a second or third salvage ASCT, an attempt to collect for at least two or more ASCTs in most patients aged under 70 years is recommended [55].

**Recommendation:** *For all ASCT eligible MM patients, stem cells should be collected within 6 months of initiating therapy especially in the presence of lenalidomide exposure*. (**Level of Evidence:** II, **Grade of Recommendation:** A)

**Recommendation:** *An attempt to collect stem cells for at least two or more ASCTs should be considered in all eligible MM patients aged under 70 years*. **(Level of Evidence:** II, **Grade of Recommendation:** A)

## Conditioning regimen

The current standard conditioning for patients with MM undergoing ASCT has been melphalan 200 mg/m^2^ ever since it was shown to be superior to melphalan 140 mg/m^2^ plus total body irradiation [65]. Unfortunately, most patients relapse post-ASCT despite receiving melphalan 200 mg/m^2^ and hence further improvements in the conditioning regimens are required with the hope of deepening response and delaying disease relapse [66]. Investigators have evaluated other conditioning regimens that combine melphalan with standard chemotherapy or novel agents with limited success [67–70]. In an open label, randomized phase III study by the IFM network, the addition of bortezomib to high-dose melphalan as part of conditioning therapy was not associated with a significantly different depth of hematologic response, PFS and OS when compared to high-dose melphalan alone [71]. Nevertheless, recently, encouraging results from a phase III trial showed that the conditioning regimen of melphalan–busulfan was safe and associated with an improved PFS (65 months vs. 34 months) compared to melphalan alone [72].

With the inclusion of novel agents such as PIs as part of induction therapy, there has been a reduction in the number of newly diagnosed MM patients still experiencing significant renal dysfunction. This is important since MM patients with elevated creatinine are at risk for higher transplant associated morbidity and mortality as well as a shorter OS [73]. Nevertheless, ASCT utilizing high-dose melphalan as the conditioning regimen can still be performed safely in MM patients with certain comorbidities, including renal insufficiency or while on hemodialysis [74–76]. Administration of reduced doses of melphalan such as 140 mg/m^2^ should be considered for all patients who are dialysis dependent or at least have a significantly impaired creatinine clearance (<30 ml/min) [77]. Patients over 70 years of age may require a reduction of the melphalan dose but this is less clear and requires clinical judgement based on the physiologic fitness, the presence of comorbidities, and the aggressiveness of the disease [34].

**Recommendation:** *High-dose melphalan chemotherapy remains the standard conditioning therapy used outside of a clinical trial. However, participation in clinical trials looking at improving the efficacy of conditioning chemotherapy in MM with novel agents is preferred if available*. (**Level of Evidence:** I, **Grade of Recommendation:** A)

MM patients with extensive extramedullary disease have poor outcomes. Retrospective results suggest that the BEAM (dexamethasone with carmustine, etoposide, cytarabine, and melphalan) conditioning that is typically used for lymphoma patients undergoing an ASCT may be effective in those MM patients with extramedullary disease [78]. There seems to be a rationale for treating such MM patients with lymphoma-like tumor biology with traditional lymphoma regimens. However, RCTs comparing BEAM to high-dose melphalan alone as conditioning therapy are lacking in MM patients with extramedullary disease.

**Recommendation:** *In those MM patients with extensive extramedullary disease undergoing an ASCT, a combination of carmustine, etoposide, cytarabine, and melphalan (BEAM) may be considered as the conditioning therapy*. (**Level of Evidence:** IV, **Grade of Recommendation:** B)

## Role for tandem ASCT

A pre-planned second ASCT within 6 months of the first ASCT meets the definition of a tandem ASCT approach. The rationale for its use is that a second exposure to high-dose melphalan may lead to a deeper hematological response. The first trial to evaluate the role of tandem ASCT in MM was in the form of Total Therapy 1 designed by the Arkansas Myeloma Group [79]. Several trials since then were conducted to compare single versus tandem ASCT. Several phase III studies demonstrated the ability of tandem ASCT to improve EFS but only one had OS improvement which was restricted to those patients who have not achieved a VGPR after the first ASCT [80–82]. These studies were performed in the era prior to novel agent induction therefore limiting the applicability of these studies to current practice. Similarly, two prior meta-analyses of studies utilizing non-novel agent treatment regimens did not show any improvement in EFS or OS [83, 84]. To address this drawback, an unplanned, pooled analysis of four different phase III studies in which single or tandem ASCT was utilized (based on the treatment center) after treatment with a bortezomib-based induction regimen was evaluated [85]. This analysis demonstrated a significant improvement in the median PFS as well as 5-year OS in favor of tandem ASCT, especially for those patients with high-risk cytogenetics such as t(4;14) and/or deletion 17p who had not achieved a CR after induction therapy (70% vs. 17%) [85]. However, since single versus tandem transplant was not decided by randomization, an element of potential bias has to be kept in mind. Therefore, these findings cannot be considered conclusive. Similarly, preliminary assessments in the EMN02/HO95 MM study showed significant improvement in the 3-year PFS (73% vs. 64%) and 3-year OS (89% vs. 82%) in favor of tandem ASCT compared to single ASCT [86]. The superiority of a tandem ASCT was especially evident in patients with high-risk cytogenetics in both 3-year PFS and OS [86]. In contrast to these positive results, the prospective phase III BMT-CTN 0702 STAMINA study showed no improvement in outcomes between patients who after an initial ASCT underwent consolidation with either (a) tandem ASCT followed by lenalidomide maintenance versus (b) consolidation with four cycles of bortezomib, lenalidomide, and dexamethasone followed by lenalidomide maintenance versus (c) lenalidomide maintenance alone [87]. Potential reasons for lack of benefit seen in the BMT-CTN 0702 trial could be that most patients received a combination of PI and IMiD-based induction therapy with variable durations lasting up to 12 months compared to the aforementioned European studies in which only the PI, bortezomib, was included in the induction therapy that was planned for a shorter duration prior to ASCT. Furthermore, the criteria for classification as high-risk disease was different between the EMN02 and BMT-CTN 0702 trials where the latter trial included patients with an elevated β2-microglobulin as having high-risk disease making it difficult to discern whether the tandem ASCT approach would have a select benefit in patients with high-risk disease defined by cytogenetic risk alone. Despite these discordant results summarized in Table 3, the role of an upfront tandem ASCT should be considered in MM patients with high-risk disease after a detailed discussion especially if the patient has not achieved at least a VGPR after the first ASCT or in the context of a clinical trial.

**Table 3:** Summary of phase III clinical trials evaluating the efficacy of tandem ASCT compared to single ASCT

Clinical trial	No.	Induction	Randomized groups	EFS/PFS	OS
Salwender/Cavo et al. [85]. Pooled analysis of 4 RCTs in NDMM	606	Bortezomib-regimen	ASCT × 1 (Not randomized)	Median PFS: 38 mos	5-year OS: 63%
		Bortezomib-regimen	ASCT × 2 (Not randomized)	Median PFS: 50 mos	5-year OS: 75%
Stadtmauer et al. [87]. NDMM pts <71 y/o	758	Any induction regimen as per clinician choice up to 12 cycles	Mel200 mg/m^2^ →R-maint	Median PFS: 52 mos	Median OS: 83 mos
			Mel200 mg/m^2^ × 2 →R-maint	Median PFS: 57 mos	Median OS: 86 mos
			Mel200 mg/m^2^ →VRd →R-maint	Median PFS: 57 mos	Median OS: 82 mos
Cavo et al. [86]. NDMM pts ≤65 y/o	1503	VCd × 3–4 cycles	Mel200 mg/m^2^ × 1 →+/-VRd →R-maint	3-year PFS: 64%	3-year OS: 82%
			Mel200 mg/m^2^ × 2 →+/-VRd →R-maint	3-year PFS: 73%	3-year OS: 89%

*R-maint* lenalidomide maintenance, *VRd* bortezomib, lenalidomide, and dexamethasone, *VCd* bortezomib, cyclophosphamide, and dexamethasone, *PFS* progression-free survival, *OS* overall survival

**Recommendation:** *In select patients with high-risk disease and good performance status, a tandem ASCT within 6 months of the first ASCT should be considered*. (**Level of Evidence:** I, **Grade of Recommendation:** C)

## Consolidation post-ASCT

The goal of consolidation therapy post-ASCT is to suppress any residual disease (i.e., deepen the hematologic response) and subsequently prolonging the duration of the response and OS while minimizing toxicity. Several RCTs have demonstrated the ability of consolidation regimens to deepen responses as well as prolong PFS; however, the impact on OS has been inconsistent [88–90]. The prospective phase III BMT-CTN 0702 STAMINA trial showed no added benefit of consolidation therapy with four cycles of bortezomib, lenalidomide, and dexamethasone in terms of PFS and OS [87]. However, the EMN02 study suggested a significant PFS benefit after consolidation with two cycles of bortezomib, lenalidomide, and dexamethasone therapy but OS benefit is unclear at present [91].

**Recommendation:** *Consolidation therapy post-ASCT is not routinely supported except in the setting of a clinical trial or in special clinical circumstances*. (**Level of Evidence:** II, **Grade of Recommendation:** B)

## Maintenance post-ASCT

In contrast to consolidation therapy, maintenance therapy is typically low-dose therapy administered over a long period of time with minimal toxicity. Single agent thalidomide has consistently shown improvements in depth of response and PFS [92–97], and in one trial [93] and two separate meta-analyses [97, 98], it also improved OS. However, clinically significant peripheral neuropathy and fatigue prevent it from being an ideal maintenance agent. Furthermore, in patients with high-risk cytogenetics such as deletion 17p, thalidomide maintenance has been associated with worse outcomes compared to placebo [99].

**Recommendation:** *Post-ASCT thalidomide maintenance is not recommended in MM patients with high-risk cytogenetics*. (**Level of Evidence:** II, **Grade of Recommendation:** B)

Maintenance with the next generation IMiD, lenalidomide, is generally better tolerated than thalidomide. Two large placebo-controlled RCTs by the IFM and CALGB demonstrated that lenalidomide maintenance deepens the hematological response as well as extends PFS significantly compared to no maintenance [100, 101]. A third prospective trial by the GIMEMA group demonstrated that lenalidomide maintenance improved median PFS compared with no maintenance [20]. However, only in the CALGB study did the extended PFS translate into an improvement in OS for patients receiving lenalidomide maintenance. A patient level pooled meta-analysis compiling all the three aforementioned RCTs confirmed the PFS benefit and demonstrated an improved OS with lenalidomide maintenance after ASCT when compared with placebo or observation [102]. However, PFS and OS benefit for lenalidomide maintenance was limited to those patients without adverse risk cytogenetics. The Myeloma XI trial which compared lenalidomide maintenance to no maintenance post-ASCT also demonstrated significant improvement in PFS with lenalidomide maintenance [103]. Finally, patients need to be advised on the small but consistent risk of secondary primary malignancies associated with prolonged lenalidomide maintenance post-ASCT [104].

**Recommendation:** *Post-ASCT lenalidomide maintenance should be considered in all MM patients with standard risk cytogenetics*. (**Level of Evidence:** I, **Grade of Recommendation:** A)

Post-ASCT maintenance with a PI such as bortezomib improved both PFS and OS for those patients with deletion 17p compared to maintenance with thalidomide outcomes reported in the HOVON-65/GMMG-HD4 trial [31, 99]. Thus, PI maintenance post-ASCT should be considered in high-risk MM patients with deletion 17p and this can likely be extended to other high-risk cytogenetic abnormalities such as t(4;14), t(14;16), and +1q amplification.

**Recommendation:** *Maintenance therapy post-ASCT with a PI should be considered in all MM patients with high-risk cytogenetics*. (**Level of Evidence:** II, **Grade of Recommendation:** B)

## Second ASCT for salvage therapy

Most patients will relapse despite consolidation and maintenance therapy post-ASCT [66]. A second ASCT as a salvage option in relapsed disease is a viable option for some of these patients. Retrospective studies, mostly from single institutions with small sample sizes and variable post-ASCT maintenance have consistently shown that a salvage second ASCT can be a safe and a beneficial option (Table 4) [105–120]. A prospective, randomized phase III study of salvage second ASCT was compared to conventional chemotherapy with cyclophosphamide and showed that the salvage ASCT improved PFS but not OS [121]. Generally, the length of PFS gained after the first ASCT is associated with the length of PFS gained after the second ASCT for salvage therapy. However, the PFS after the second ASCT as salvage therapy is generally shorter than that gained after the first ASCT.

**Table 4:** Retrospective studies evaluating the use of salvage ASCT in relapsed multiple myeloma

Study	No.	ORR (%)	Median PFS (months)	Median OS (months)	TRM (%)
Shah et al. [111]	44	90	12.3	31.7	2
Jimenez-Zepaeda et al. [110]	81	97	16.4	53	3
Olin et al. [116]	41	55	8.5	20.7	7
Fenk et al. [114]	55	75	14	52	5
Alvares et al. [120]	83	—	15.6	34.8	—
Burzynski et al. [115]	25	64	12	19	8
Mehta et al. [133]	42	81	12.5	32	10
Eliece et al. [117]	26	69	14.8	38.1	0
Gonsalves et al. [107]	98	86	10.3	33	4
Yhim et al. [119]	48	—	18	55.5	—
Lemieux et al. [108]	81	93	18	48	0
Michaelis et al. [109]	187	—	3-year PFS: 13%	3-year OS: 46%	2

*TRM* treatment-related mortality, *ORR* overall response rate, *PFS* progression-free survival, *OS* overall survival

**Recommendation:** *Second ASCT as salvage therapy should be considered in patients with MM who had adequate duration of disease control with their first ASCT:* *>* *18 months unmaintained or* *>* *36 months-maintained response to first ASCT*. (**Level of Evidence:** II, **Grade of Recommendation:** B)

## Allogeneic stem cell transplantation

Even though evidence of a graft versus myeloma effect associated with long-term disease control or cure has been established in MM, the role of an allogeneic stem cell transplant (Allo-SCT) in MM remains controversial. Allo-SCT can be performed with either myeloablative or reduced intensity conditioning (RIC). Unfortunately, myeloablative approaches have been fraught with significant treatment-related mortality (TRM) [122–124] and hence RIC allo-SCTs have been favored. The latter offers the ability for less toxic conditioning but with the benefits of a continuous graft versus myeloma effect. Several studies have evaluated tandem ASCT versus ASCT followed by RIC Allo-SCT in the upfront setting; however, the pre-transplant induction therapy, conditioning regimens, and graft versus host disease prophylaxis are quite different among these trials making cross-trial comparisons difficult (Table 5) [125–130]. Nevertheless, with long follow-up, some studies have suggested a benefit for the ASCT-RIC-allo-SCT approach [127, 129]. However, high TRM remains an issue and in a meta-analysis of all available studies, there was a lack of benefit of ASCT-RIC-allo-SCT over a tandem ASCT [131]. Given these inconsistent and contradictory results, the use of an ASCT-RIC-allo-SCT in the upfront setting is mostly limited to the setting of a clinical trial. The assessment of an Allo-SCT with RIC conditioning in the relapsed setting has been limited to retrospective studies. However, it has been uniformly demonstrated that MM patients with multiply relapsed disease do not appear to obtain any significant survival benefit from a salvage allo-SCT compared to salvage ASCT despite some studies suggesting lower relapse rates in the former [118, 132–137]. This lack of survival benefit with a salvage Allo-SCT seems to be driven by a higher rate of non-relapse mortality. Thus, allo-SCT should not be done as a “last resort”. If contemplated, it should be done earlier in the disease course, for carefully selected patients with “high-risk disease”, ideally on a clinical trial. More importantly, patients must be made aware and be willing to accept the risk of toxicities associated with allo-SCT such as graft versus host disease and non-relapse mortality.

**Table 5:** Summary of phase III clinical trials evaluating the efficacy of tandem ASCT to ASCT-RIC-Allo-SCT in the upfront setting for patients with MM

Clinical trial	No.	Randomized groups	ORR	EFS/PFS	OS	TRM
Krishnan et al. [130]. NDMM pts ≤70 y/o	710	ASCT × 2	CR: 45%	3-year PFS: 46%	3-year OS: 80%	3-year TRM: 4%
		ASCT→RIC-Allo-SCT (TBI 2 Gy)	CR: 58%	3-year PFS: 43%	3-year OS: 77%	3-year TRM: 11%
Giaconne et al. [129]. NDMM pts ≤65 y/o	162	ASCT × 2	CR: 26%	Median EFS: 2.4 years	Median OS: 4.25 years	2-year TRM: 2%
		ASCT→RIC-Allo-SCT (TBI 2 Gy)	CR: 55%	Median EFS: 2.8 years	Median OS: NR	2-year TRM:10%
Gahrton et al. [127]. NDMM pts <70 y/o	357	ASCT × 2	CR: 41%	8-year PFS:12%	8-year OS: 36%	3-year TRM: 3%
		ASCT→RIC-Allo-SCT (Flu/TBI 2 Gy)	CR: 50%	8-year PFS: 22%	8-year OS: 49%	3-year TRM: 13%
Moreau et al. [128]. NDMM pts <65 y/o (w/ del 13 and β2-microglobulin >3 mg/l)	284	ASCT × 2 (+/− anti-IL6 antibody)	CR: 38%	Median EFS: 22 mos	Median OS: 48 mos	5-year TRM: N/A
		ASCT→RIC-Allo-SCT (Flu/Bu)	CR: 62%	Median EFS: 19 mos	Median OS: 34 mos	5-year TRM: 11%
Rosinol et al. [126]. NDMM pts <70 y/o	752	ASCT × 2	CR: 11%	Median PFS: 31 mos	Median OS: 58 mos	TRM: 5%
		ASCT→RIC-Allo-SCT (Flu/Mel)	CR: 40%	Median PFS: NR	Median OS: NR	TRM: 16%
Knop et al. [125]. NDMM pts ≤60 y/o (w/ del 13)	199	ASCT × 2	CR: 31%	Median PFS: 23 mos	Median OS: 72 mos	2-year TRM: 4%
		ASCT→RIC-Allo-SCT (Flu/Mel)	CR: 59%	Median PFS: 35 mos	Median OS: 70 mos	2-year TRM: 12%

*ORR* overall response rate, *EFS* event-free survival, *PFS* progression-free survival, *OS* overall survival, *CR* complete remission, *TRM* transplant-related mortality, *NR* not reached

**Recommendations:** *We do not recommend the routine use of a myeloablative or RIC-Allo-SCT as upfront therapy except in the setting of a clinical trial or in special clinical circumstances*. (**Level of Evidence:** I, **Grade of Recommendation:** C)

**Recommendations:** *We suggest considering the use of Allo-SCT as salvage therapy in eligible patients younger than 60 years of age, with high-risk disease and who have experienced an early relapse after primary therapy, but preferably in the setting of a clinical trial*. (**Level of Evidence:** I, **Grade of Recommendation:** C)

## Conclusions and future directions

The field of MM has seen unprecedented success as the survival of patients with MM has improved considerably over the last two decades with the incorporation of highly effective novel therapies. However, even though the extent of benefit provided by ASCT and its timing will continue to be debated in the era of continued development of novel therapies, it is still clear that ASCT improves the depth of response and provides significant PFS benefit producing unprecedented survival outcomes. Thus, ASCT remains an integral part of the management plan of all newly diagnosed MM patients who are otherwise eligible. Similar to our current Mayo Clinic consensus on the use of HSCT in MM, the European Myeloma Network (EMN) guidelines also support the use of an upfront ASCT with high-dose melphalan in all eligible MM patients followed by PI and/or IMiD-based maintenance therapy [138]. However, differences between the two guidelines exist in the strength of recommendation for routine use of consolidation therapy for all MM patients prior to maintenance therapy. Nevertheless, both the Mayo Clinic and EMN guidelines strongly recommend the use of salvage ASCT for relapsed disease based on the duration of disease control obtained after the first ASCT. Finally, the upfront use of an allo-SCT is not recommended by either consensus guidelines except in the setting of a clinical trial or in special clinical circumstances.

The standardization and availability of MRD testing for clinical use could allow for a more refined application and identification of sub-groups of patients who are most likely to benefit or for whom ASCT can be safely deferred to first relapse. Just like the standard treatment of MM, ASCT is not static. Novel conditioning regimens are being evaluated that incorporate novel agents (carfilzomib) or traditional chemotherapy drugs to build on the efficacy of melphalan. There appears to be a role for the use of tandem ASCT in MM patients with high-risk cytogenetics. However, the role for allo-SCT still needs to be better defined in the context of MM. Finally, the role of ASCT will continue to have to be defined in the context of emerging immunotherapies such as mABs, antibody drug conjugates, immune checkpoint inhibitors, Bispecific T-cell Engagers (BiTe), and chimeric antigen receptor-T cell therapy enter into clinical practice. ASCT should be seen as complementary rather than in competition to other available treatments, and a judicious, personalized use of the right treatment at the right time can be expected to lead to the best outcome for the patient.

## Electronic supplementary material

Supplement Table 1
