# Supplementary material for: Utilization of hematopoietic stem cell transplantation for the treatment of multiple myeloma: a Mayo Stratification of Myeloma and Risk-Adapted Therapy (mSMART) consensus statement
Source: Bone Marrow Transplant. 2018 Jul 9;54(3):353–67. doi: 10.1038/s41409-018-0264-8 (PMC6463224; doi:10.1038/s41409-018-0264-8)
Supplement: Supplementary file 1 — Supplement Table 1 [file 41409_2018_264_MOESM1_ESM.docx]

**SUPPLEMENT** **TABLE 1:** Summary of clinical trials evaluating the efficacy of ASCT in the era prior to novel agent-based induction therapy

| **Study** | **No** | **Induction** | **Randomized arms** | **ORR** | **EFS/PFS** | **OS** |
| --- | --- | --- | --- | --- | --- | --- |
| **Attal et al.**^12^  NDMM pts < 65 y/o | 200 | VMCP/ BVAP x 4-6 cycles | Continued VMCP/ BVAP up to 18 cycles | >VGPR: 14% | Median EFS: 18 mos  5-yr EFS: 10% | Median OS: 37 mos  5-yr OS: 12% |
|  |  |  | Mel140 mg/m2 + 8Gy TBI | >VGPR: 38%^*^ | Median EFS: 27 mos^*^  5-yr EFS: 28%^*^ | Median OS: NR^*^  5-yr OS: 52%^*^ |
| **Child et al.**^13^  NDMM pts < 65 y/o | 407 | Doxorubicin / Carmustine / Melphalan / Cyclophosphamide x at least 3 cycles | up to 12 cycles | CR: 8% | Median PFS: 20 mos | Median OS: 42 mos |
|  |  |  | Mel200 mg/m2 | CR: 44%^*^ | Median PFS: 32 mos^*^ | Median OS: 54 mos^*^ |
| **Ferdinand el al.**^14^  NDMM pts 55-65 y/o | 190 | VMCP or VAMP x 3-4 cycles | Continued VMCP or VAMP | CR+MRD: 20% | Median EFS: 19 mos | Median OS: 48 mos |
|  |  |  | Mel200 mg/m2  or  Mel140 mg/m2 + Busulfan (4 mg/Kg/d on days -6 to -3) | CR+MRD: 36%^*^ | Median EFS: 25 mos | Median OS: 48 mos |
| **Barlogie et al.** ^15^  NDMM pts < 70 y/o | 516 | VAD x 4 cycles | VBMCP for up to 1-year | N/A | 7-yr PFS: 14% | 7-yr OS: 38% |
|  |  |  | Mel140 mg/m2 + 12Gy TBI | N/A | 7-yr PFS: 17% | 7-yr OS: 38% |
| **Blade et al.**^16^  NDMM pts < 65 y/o | 216 | VBMCP/VBAD x 4 cycles | Continued VBMCP/VBAD x 8 cycles | CR: 11% | Median PFS: 33 mos | Median OS: 61 mos |
|  |  |  | Mel200 mg/m2  or  Mel140 mg/m2 + 12Gy TBI | CR: 30%^*^ | Median PFS: 42 mos | Median OS: 66 mos |
| **Palumbo et al.**^17^  NDMM pts 50-70 y/o | 194 | MP  or  VAD x 2 cycles | MP x 6 cycles | nCR: 6% | Median EFS: 16 mos | Median OS: 43 mos |
|  |  |  | Mel100 mg/m2 x 2 | nCR: 25%^*^ | Median EFS: 28 mos^*^ | Median OS: NR^*^ |

CR: Complete response

nCR: Near complete response

EFS: Event free survival

PFS: Progression free survival

OS: Overall survival

VAD: Vincristine, doxorubicin and dexamethasone

MP: Melphalan and prednisone

VBMCP: Vincristine, carmustine, melphalan, cyclophosphamide and prednisone

VAMP: Vincristine, doxorubicin and methylprednisone

VBAD: Vincristine, BCNU, doxorubicin and dexamethasone

NR: Not reached
